# Hepatitis Delta Virus: Replication Strategy and Upcoming Therapeutic Options for a Neglected Human Pathogen

**DOI:** 10.3390/v9070172

**Published:** 2017-07-04

**Authors:** Florian A. Lempp, Stephan Urban

**Affiliations:** 1 Department of Infectious Diseases, Molecular Virology, Heidelberg University, Im Neuenheimer Feld 345, 69120 Heidelberg, Germany; Florian.Lempp@med.uni-heidelberg.de; 2 German Centre for Infection Research (DZIF), Partner Site Heidelberg, 69120 Heidelberg, Germany

**Keywords:** Hepatitis D virus, lonafarnib, REP2139-Ca, Myrcludex B, viroid

## Abstract

The human Hepatitis Delta Virus (HDV) is unique among all viral pathogens. Encoding only one protein (Hepatitis Delta Antigen; HDAg) within its viroid-like self-complementary RNA, HDV constitutes the smallest known virus in the animal kingdom. To disseminate in its host, HDV depends on a helper virus, the human Hepatitis B virus (HBV), which provides the envelope proteins required for HDV assembly. HDV affects an estimated 15–20 million out of the 240 million chronic HBV-carriers and disperses unequally in disparate geographical regions of the world. The disease it causes (chronic Hepatitis D) presents as the most severe form of viral hepatitis, leading to accelerated progression of liver dysfunction including cirrhosis and hepatocellular carcinoma and a high mortality rate. The lack of approved drugs interfering with specific steps of HDV replication poses a high burden for gaining insights into the molecular biology of the virus and, consequently, the development of specific novel medications that resiliently control HDV replication or, in the best case, functionally cure HDV infection or HBV/HDV co-infection. This review summarizes our current knowledge of HBV molecular biology, presents an update on novel cell culture and animal models to study the virus and provides updates on the clinical development of the three developmental drugs Lonafarnib, REP2139-Ca and Myrcludex B.

## 1. Introduction

Hepatitis Delta Virus (HDV), the only member of the genus *Deltavirus* and the causative agent for chronic Hepatitis D (CHD), represents the smallest known animal virus and shows peculiar characteristics in both its morphology and replication cycle. HDV is a satellite virus/virusoid of the human Hepatitis B Virus (HBV) as it requires the HBV envelope proteins (HBsAg) to form virus particles. Accordingly, HDV infection either establishes as a superinfection of an HBV-carrier or by simultaneous contact with HBV and HDV (see Table 1 for a characterization of both viruses). Originally discovered in 1977 [1], HDV is still a neglected pathogen, although it causes the most severe form of viral hepatitis. Presently, about 15–20 million people worldwide are chronically infected with HDV, which is more than half the number of HIV-infected individuals. In contrast to HIV, these patients still lack appropriate treatment options and show a pronounced reduction in their life expectation [2,3]. In this review, we summarize the current knowledge about the HDV molecular virology, HDV infection models in research, global epidemiology and finally provide an outlook of current investigational antiviral drugs in clinical development.

## 2. Molecular Virology

### 2.1. Virus Structure

**Figure 1:**
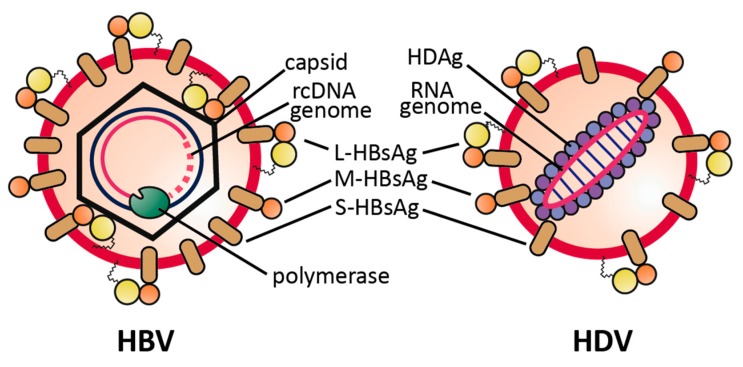
Schematic representation of HBV and HDV virions. Both virions share the same envelope proteins, the S-, M- and L-HBsAg. L-HBsAg is composed of S-HBsAg (brown) with two *N*-terminal elongations: preS2 (orange) and the *N*-terminally myristoylated preS1 (yellow).

The Hepatitis Delta virion comprises a circular, single-stranded, negative sense RNA, composed of 1672–1697 nucleotides, dependent on the genotype. The genome is viroid-like, due to a high degree (~74%) of intramolecular base pairing [4,5,6]. Both forms of the virally-encoded Hepatitis Delta Antigen (HDAg), small (S-)HDAg and large (L-)HDAg, are associated with the genomic RNA (having negative polarity), thereby forming the viral ribonucleoprotein (RNP) complex. This RNP becomes enveloped by a lipid bilayer derived from the host endoplasmic reticulum, carrying the three HBV envelope proteins small (S-)HBsAg, middle (M-)HBsAg and large (L-)HBsAg to assemble into an infectious HDV particle Figure 1.

Envelope proteins and RNP associate via a lipid farnesyl-moiety on the L-HDAg that directly binds to a cytoplasmic loop of the S-HBsAg. In total, the HD virion, which measures 35–37 nm in diameter, is slightly smaller than the HBV particle with 42 nm but larger in diameter than the HBV 22 nm subviral particles (spheres, filaments) that are found in excess in the serum of HBV-infected patients [7].

### 2.2. Viral Replication Cycle

HDV and HBV show a distinct liver tropism by usage of the same envelope proteins and consequently share two essential host factors for viral entry. After entering the Space of Dissé, the virions attach to cellular heparan-sulfate proteoglycans (HSPGs), mediated by both the preS1 domain of the viral L-HBsAg and the antigenic loop of the S-domain [8,9,10,11]. Some evidence suggests that Glypican-5 is a major but not the only core HSPG for attachment [12]. After HSPG interaction, the virion switches to its hepatocyte-specific receptor human sodium taurocholate cotransporting polypeptide (hNTCP) and irreversibly binds via the myristoylated *N*-terminal preS1 region of the L-HBsAg [13,14]. As a hepatocyte-specific bile salt transporter with basolateral localization, hNTCP not only mediates liver tropism of both HBV and HDV but also plays an important role in species-specificity of the two viruses, since only the hNTCP but not NTCP homologues from mouse, rat, macaque or pig, support virus entry [13,15,16,17]. While mouse and rat NTCP still support binding to the HBVpreS-domain, pig and cynomolgus NTCP are completely deficient in preS-binding [15], a fact that could be related to distinct species-specific sequence diversities within the respective NTCP proteins [13]. HDV is taken up into the hepatocyte by so far ill-characterized processes and the viral membrane fuses most probably with the endosomal membrane to release the RNP. Once in the cytoplasm, the genomic RNP complex is transported to the nucleus, guided by a nuclear localization signal within the HDAg [18,19]. RNA replication occurs exclusively in the nucleus and is mediated by host DNA-dependent RNA polymerases (probably RNA pol II), as HDV does not encode its own RNA-dependent RNA polymerase [20,21]. During a first rolling-circle replication strategy, multimers of antigenomic RNA sequences are transcribed and cleaved to uniform one-unit length by the viral ribozyme encoded in the antigenomic strand [22,23]. Circularization of the monomers is mediated by the ribozyme but also cellular ligases might play a role. In the second rolling-circle replication, multimers of genomic polarity a transcribed using the antigenomic templates and cleaved by a second viral ribozyme encoded in the genomic strand. The HDV genome contains one open reading frame (ORF), which is located on the antigenomic strand and encodes the S-HDAg. Translation of S-HDAg, however, does not occur from the antigenomic RNA, as this is located exclusively in the nucleus, but from a 5′-capped and 3′-polyadenylated ~800 nt mRNA that is transcribed and exported into the cytoplasm [24,25,26]. During replication, the antigenomic RNA becomes edited by the cellular enzyme Adenosine Deaminase Acting on RNA (ADAR1), which catalyzes the mutation of the S-HDAg ORF stop codon (UAG) to a tryptophan codon (UGG) leading to the 3′ elongation of the ORF [27,28,29]. The resulting mRNA leads to L-HDAg protein comprising an S-HDAg with a 19/20 amino acid *C*-terminal elongation (genotype dependent). This extension contains a nuclear export signal and an HBsAg binding motif through a prenylation site at Cys211, which is post-translationally farnesylated by a cellular farnesyltransferase [30,31]. Farnesylated L-HDAg downregulates viral replication, drives the viral replication cycle towards assembly and is required for assembly as it interacts with the cytosolic loop of the HBsAg during envelopment [32,33]. As replication proceeds, genomic RNA in complex with HDAg is exported from the nucleus, probably by nuclear export factor 1 (TAP) and the cellular RNA export factor REF/Aly [34]. Assembly of HDV particles can only take place in HBV co-infected cells, as HBsAg is required for envelopment. However, HBsAg might also be provided by defective HBV genomic integrates that do not encode full HBV pregenomic RNA but still encode self-assembly competent HBV envelope proteins [35]. Functional, infectious particles are enveloped with both L-HBsAg and S-HBsAg. In principle, only the S-HBsAg is required for HDV envelopment (in contrast to HBV, which requires also L-HBsAg), however, as the L-HBsAg is crucial for infectivity, particles enveloped in only S-HBsAg remain non-infectious although they may contribute to the “viral load” in infected patients [36,37]. Enveloped particles are subsequently secreted from cells. Whether this occurs via the classical secretion pathway (like for SVPs) or via multivesicular bodies (like for HBV) remains unclear. See Lempp et al. [38] for a schematic representation of the viral replication cycle.

## 3. HDV Infection Models

### 3.1. In Vitro Infection Models

Before the discovery of the cellular receptor hNTCP, cell culture models for HDV were limited either to transfection based systems or infection of HepaRG cells and primary human hepatocytes (PHH), which are scarce in availability and show broad heterogeneity between donors [39,40,41]. Common liver cell lines like HuH7 and HepG2 are resistant to HDV infection as they lost NTCP expression. After the cloning and sequencing of a complete HDV genome in the 1980s, cells transfected with HDV-encoding plasmids or naked HDV RNA were the first in vitro models to study viral replication (Table 2). Both liver and non-liver cell lines replicate HDV when transfected with a HDV cDNA plasmid [42]. When cells are transfected with in vitro transcribed HDV RNA, only those cells that additionally express S-HDAg are able to replicate HDV, emphasizing the importance of HDAg for HDV replication [43]. Cells transfected with both plasmids coding the HDV cDNA as well as plasmids coding the HBV surface proteins, replicate HDV and secrete infectious particles [44,45]. Functional particles can even be assembled with HBsAg derived from a natural stable HBV integrate when HDV cDNA is transfected [35].

Initially isolated from an HCV-infected patient, the HepaRG cell line was the first immortalized cell line that showed susceptibility to HBV and HDV infection. During a 4-week differentiation protocol, biliary cells and hepatocyte-like cells are formed that present many characteristics of fully differentiated hepatocytes including polarization, expression of hepatocyte markers and formation of bile canaliculi [46]. HepaRG cells have been used to elucidate the viral entry process [8,41], to analyze drug candidates and to analyze innate immune activation during HDV infection [52]. The identification of hNTCP lead to the stable reconstitution of liver-derived hepatoma cell lines expressing the receptor (HepG2-hNTCP, HuH7-hNTCP), thereby becoming susceptible to both HBV and HDV [13]. These cell lines are easy-to-handle, show susceptibility to HDV infection at rates up to 30% and even support innate immune activation to some extent (e.g., for HepG2 cells). All hNTCP-expressing cell lines are susceptible to HDV mono-infection. The virus can enter the cells, start replication and express HDAg in the nucleus. Subsequent steps including assembly and release of progeny virus are blocked as they lack HBsAg. HepG2 cells stably expressing both hNTCP and HBsAg (large and small) support the full HDV replication cycle including progeny release, which can be quantified by re-infection experiments. These cell lines, which are currently being developed, are suitable for high-content screening approaches as all steps within the viral life cycle can be addressed [53]. More recently, stem-cell derived hepatocytes have been described as a novel HBV infection system. These hepatocytes are differentiated in vitro from either embryonic or induced pluripotent human stem cells. After differentiation, the cells endogenously express NTCP and become susceptible to HBV infection at similar levels than PHH [47,48,49,50,51]. Susceptibility to HDV has not been shown yet but is likely. A major advantage of stem-cell derived hepatocytes compared to PHH is the reproducibility and the possibility to obtain them from donors with defined polymorphisms of interest. However, differentiation protocols are complex and time-consuming and ethical restrictions apply in some countries considering the work with embryonic stem cells.

### 3.2. In Vivo Infection Models

HDV, similar to HBV, exhibits a narrow host range, naturally infecting only members of the great apes (human, chimpanzee) but not common laboratory animals like mice, rats or macaques. Accordingly, the chimpanzee has been the primary in vivo HDV infection model for transmission studies and the analysis of infectivity of cloned HDV cDNA [54,55,56] (Table 3). Although it remains the only immunocompetent animal model for HDV and HBV, all experiments with chimpanzees for biomedical research have been stopped due to ethical considerations. Remarkably, the eastern woodchuck can be infected at a low level with HDV [57]. This early observation is consistent with the recent observation that woodchuck NTCP supports low levels of HDV infection [58]. When co-infected with the Woodchuck Hepatitis Virus (WHV), a relative of HBV within the genus *orthohepadnaviruses*, the replicating HDV RNP can be packaged into the WHV envelope and secreted into the serum, where it infects neighboring hepatocytes with much higher efficiency than the initial HDV. Although this model is suitable to address some specific questions, e.g., related to the development of HDAg-specific therapeutic vaccines [59], it relies on WHV and not HBV. One major difference between both viruses is that the WHV envelope proteins probably recognize a receptor different from hNTCP and therefore the model cannot be applied to study the natural infection/co-infection of HBV and HDV. Transgenic mouse models were generated that encode in their genomes either the HDAg ORF [60] or carry integrates with a replication-competent dimer of the full HDV genomic sequence [61]. Neither HDAg-expression nor replicating HDV led to the development of hepatitis or showed any histopathological evidence for disease. An interesting aspect of the latter study was the notion that the HDV genome was able to replicate in many different tissues with the highest replication levels not even in the liver but in the skeletal muscle, indicating that the natural tissue tropism of HDV is predominantly mediated by the HBV-envelope proteins. Liver specific HDV replication can also be achieved by hydrodynamic injection of plasmids or naked RNA encoding the HDV genome [62]. Through co-injection of HDV- and HBsAg- encoding plasmids, assembly and release of HDV particles can be observed. Release of particles can be inhibited by prenylation inhibitors [63]. However, viral spread does not occur in these systems because mouse NTCP does not confer susceptibility to HDV. After the discovery of hNTCP as a bona fide receptor and the finding that mouse NTCP does not support entry [13,17], transgenic mice expressing human NTCP were generated and showed low-level susceptibility to HDV (but still no susceptibility to HBV) infection [64,65]. However, these studies also revealed several additional restraints: (i) only very young mice could be infected; (ii) high virus inocula were required and (iii) infection was transient and cleared after 20 days. Currently, the most widely used murine in vivo models are liver chimeric mice. These mice either have a genetic defect leading to a growth disadvantage of mouse hepatocytes or have a pronounced sensibility to toxic agents specifically affecting the mouse hepatocytes. This permits partial repopulation of the murine liver with HBV- and HDV-susceptible human hepatocytes [66]. All of the mice have an immune deficient genetic background (mostly SCID or NOG), which ensures that the human hepatocyte graft is not rejected. As liver chimeric mice are susceptible to both viruses, HDV monoinfection, HDV/HBV co-infection and even superinfection can be studied in this model [67], which has also shown that HDV is a potent activator of the host innate immunity [68].

Liver chimeric mice are important infection models that allow studying many aspects of the viral life cycles, except for immunopathogenesis, development of liver disease/HCC and evaluation of vaccines. However, the model is complex, depends on the availability of PHH and experiments are time consuming. Recently, we showed in vitro that NTCP is the only factor restricting HBV/HDV infection in macaque and pig hepatocytes [15,71]. NTCPs from both species do not support viral entry, however, when primary hepatocytes of macaque or pig are transduced to express hNTCP, these cells become fully susceptible to both viruses. Accordingly, hNTCP-transduced macaques and pigs could become the first immunocompetent animal models that support infection with both viruses.

## 4. Epidemiology

**Figure 2:**
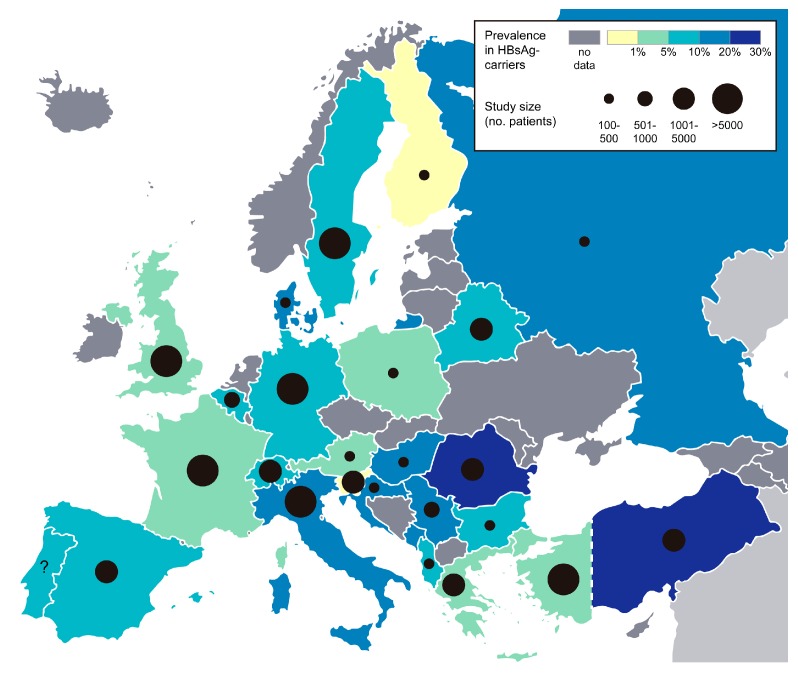
HDV prevalence in HBsAg-carriers in Europe. Prevalence of anti-HDV antibodies detected in chronic HBV carriers. Black circles indicate the number of HBV patients that were included in the respective prevalence studies (or combined number of patients, when more than one study was available). See [79] for a worldwide prevalence map. Raw map file is distributed under the Creative Commons (Attribution 3.0) license (http://freedesignfile.com).

HDV like HBV transmits via the parenteral route, through contact with infectious body fluids e.g., blood, semen or vaginal fluid. Vertical transmission from mother to child is possible but rare [72,73]. As all chronically HDV-infected patients are co-infected with HBV, prevalence data of HDV is given as percentage of HDV-positive patients in HBsAg-positive individuals. Worldwide, an estimated 5% of HBsAg-carriers are co-infected with HDV, which corresponds to ~15 million individuals. Geographical distribution varies considerably, with very high prevalence rates (>20%) in the Amazon Basin in South America, Central Africa, Iran, Pakistan, Eastern Turkey and Mongolia [74,75]. In Europe, prevalence rates range between ~1% (Finland, Slovenia) and 25% (Romania) (see Figure 2). Since the HBV vaccination programs in the 1980s, HDV prevalence rates have dropped in many European countries e.g., in Italy from 25% in 1983 to 9% in 2014 [76,77]. In Central Europe, immigration accounts for a large proportion of chronic HDV-infected patients, a study in Germany showed that 75% of HDV-positive patients have migrated from Turkey or Eastern Europe [78].

Recent data from the Unites Stated suggest HDV prevalence rates similar to those in Europe, ranging from 3–8% [80,81]. In China, a country with high HBV endemicity, HDV prevalence rates of 1–9% have been described [82,83,84,85]. Solid prevalence data from Russia are still lacking, although Russia is a country with intermediate (2–7%) HBV prevalence [86]. The few studies published so far suggest HDV prevalence rates of 5–16% [87,88,89]. A major problem of HDV diagnosis remains the low testing rates in HBV-infected individuals in different countries. In principle, every HBsAg-positive individual should be tested for the presence of anti-HDV antibodies and, if positive, for HDV RNA to confirm active viral replication. However, many hospitals do not routinely check for the anti-HDV antibody status of HBV patients and testing rates therefore vary dramatically (e.g., only 35% HBsAg-positive individuals were tested in Greece [90], only 8% in a study from the US [81] and 40–99% in the UK [91]). Reasons for the testing reluctance might be unawareness and labor-intense diagnostic tests (mostly manual ELISAs) at high costs. Moreover, the current lack of an effective therapy for chronic Hepatitis D may be another hurdle to routinely implement full coverage of HBV and HDV diagnostics.

## 5. Current Treatment Options and Drugs in Development

### 5.1. Current Treatment Options

At present treatment options for chronic Hepatitis D patients are limited to interferon-alpha (IFNα), [92] and its improved derivative pegylated IFNα (pegIFNα) [93]. Due to the advanced disease state of the liver in many HDV patients, only a minority of these are eligible for IFNα treatment. To control at least the HBV-related disease progression, nucleoside/tide analogues (NUCs) with a low barrier to resistance (e.g., tenofovir or entecavir) can be regarded as a possible treatment option for patients with considerable HBV replication [94]. Remarkably, treatment with tenofovir in HDV/HBV-infected patients co-infected with HIV showed marked reductions in HDV viremia [95]. Since NUCs do not directly affect the helper function of HBV (envelope protein expression from ccc- or integrated HBV-DNA), they cannot suppress HDV assembly in infected cells. Moreover, since these drugs specifically act on the HBV reverse transcriptase, they have no direct effect on HDV RNA replication (unpublished data). The partial efficacy of IFNα in HDV/HBV co-infected patients can be attributed to two mechanistically distinguishable modes of IFNα activity. First, IFNα can directly suppress HDV replication to some extent by a still undefined mechanism, as has been shown in in vitro studies (unpublished data) and in clinical trials [96,97,98]. Second, in a clinical context, IFN and pegIFNα can—in very rare cases—induce negativation of HBsAg, possibly through elimination of HBsAg producing hepatocytes. These might be covalently closed circular DNA (cccDNA)-positive cells or cells with integrated HBV DNA encoding HBsAg. This sustained suppressed state of HBsAg is defined as a “functional cure” of both HBV and HDV infection and is achieved in less than 1% of HBeAg negative patients under NUC treatment [99].

Approaches to combine IFNα with nucleoside analogs in order to improve the therapeutic outcome were investigated in two clinical trials. In the HIDIT-1 trial IFNα monotherapy was compared to combination of pegIFNα with adefovir (ADV)—a first generation NUC—or ADV alone for 48 weeks. Patients experienced RNA negativation in 31%, 26% and 0% of cases 24 weeks after drug withdrawal [93]. Unfortunately, 56% of these RNA negative patients relapsed during long-term follow-up indicating that HDV clearance without HBsAg-negativation cannot be accomplished [100] and a state of “sustained virological response”, as achieved after DAA-therapy in HCV-infected patients cannot be defined for HDV patients. Prolonged treatment with pegIFNα alone or in combination with tenofovir for 96 weeks (HIDIT-2 study), showed no improvement in treatment outcome but was associated with a higher frequency of IFNα-related serious adverse events [101]. Considering the severe side effects induced by IFNα, the alternative was raised of using IFN-λ for treating a subpopulation of IFNα non-eligible HDV patients. A clinical trial evaluating the activity of this cytokine has recently been initiated in HDV/HBV-co-infected patients. Nevertheless, the generally low treatment responses of IFNα and the limited eligibility of many patients for IFNα-treatment poses the challenge of developing more specific novel therapeutic approaches to durably control or, in the best case, functionally cure HDV infection. Such future therapies could principally aim at controlling HBsAg-secretion in order to control the helper function of HBV, HBV/HDV-entry to prevent the de novo formation of HBV cccDNA or HDV RNA replicative intermediates to directly or indirectly target the HDV replicative life cycle (see below).

### 5.2. Possible Targets to Interfere with HDV Replication

Antivirals affecting HDV replication can address host or virus specific factors, with the latter acting either specifically on HDV or indirectly through reduction of HBs particles release. Since host specific enzymes (like Pol-II or farnesyl transferase) catalyze many steps in the HDV replication cycle, drugs that directly act on viral intermediates may be limited and in fact have not been described so far. This lack of knowledge is also related to the fact that HDV replication systems were lacking for a long time [13,14,53]. Nevertheless, we cannot exclude that such substances might be identified through application of NTCP-based screening systems in the future [12].

#### 5.2.1. Viral factors

Since HDV uses the cellular RNA Pol II as its replication enzyme [102] the development of drugs directly targeting RNA replication is not achievable. However, the processing of the oligomeric linear HDV RNA intermediates or cleavage and ligation of the monomeric HDV RNA depends on the activity of two RNA-encoded ribozymes. Inhibition of other ribozymes by small molecules has been described before [103] and might represent a feasible way of directly affecting HDV replication. However, such inhibitors have not yet entered the clinical state. An alternative approach could be siRNA- or shRNA-mediated therapeutic silencing of HDV RNA intermediates (e.g., by targeting the mRNA of HDAg) in patients. The feasibility and efficacy of such strategies has been demonstrated in animal models of HBV and clinical trials [104,105]. However, attention has to be paid on safety issues of of the applied hepatocyte-specific delivery systems. Besides viral RNAs the large and the small HDAgs are suitable targets to interfere with HDV replication. Since both antigens participate at various steps in HDV replication (see Section 2.2) they might act through multiple modes of action. No such compounds have been developed so far.

#### 5.2.2. Host Factors

So far, only a few of probably many unknown HDV-related host factors are described [106]. Interestingly, two of the three novel therapeutic agents presently investigated in clinical trials (Myrcludex B and Lonafarnib; see below) address such factors. Myrcludex B specifically blocks the HDV/HBV receptor function of hNTCP [13,14] and in doing so, prevents virus-dependent intrahepatic spread. Lonafarnib inhibits prenylation of the L-HDAg through inhibition of Farnesyltransferease, a cellular enzyme essential for the prenylation of cellular factors (e.g., c-Ras). It thereby prevents the envelopment and release of HDV RNP-complexes.

HDV in contrast to HBV induces strong innate immune responses in infected hepatocytes [52,65,68] and in an AAV-based mouse model [70]. Activation predominantly proceeds via IFNβ and IFNλ. Recognition of HDV by the cell is achieved by MDA5 [107]. Thus, HDV evolutionary adapted to replicate in IFN-activated state raising the question by which mechanisms IFNα-therapy of HDV patients leads to a reduction of HDV RNA-serum levels. Besides activation of cellular immune response against HDV-infected cells (which obviously does not result in a complete elimination of HDV-infected cells as we know from the clinical data of IFNα-therapy), one explanation could be a non-HDV-related effect of IFNα resulting in killing of hepatocytes. Regarding future treatment approaches using IFNλ, one has to consider that this cytokine is already constantly induced by HDV itself. An advantage of using IFNλ could therefore be its improved liver specificity and its possible combination with other drugs aiming at eliminating HDV [108].

### 5.3. Novel Treatment Options for Chronic Hepatitis D Virus Infections

Three novel drugs are currently investigated in phase II trials in chronic HDV-infected patients (Table 4). (1) Lonafarnib, an orally administered prenylation inhibitor preventing the egress of enveloped HDV particles [109,110,111]. (2) Nucleic acid polymers like REP2139-Ca, which is administered intravenously and has been described to affect the HBsAg but presumably exhibits additional modes of action [112,113,114,115]. (3) Myrcludex B, a subcutaneously delivered myristoylated L-HBsAg-derived 47-mer lipopetide, which irreversibly blocks the NTCP receptor of HDV (and HBV) thereby preventing *de novo* formation of HDV RNA- and cccDNA in naïve and regenerating hepatocytes. All three drugs are being evaluated alone or in combination with pegIFNα and/or a NUC like tenofovir but—so far—not in combination with each other. Follow up trials for all three drugs are ongoing and are expected to be presented instantly in upcoming liver meetings (AASLD, EASL). Detailed results of study designs and interim results as presented at previous meetings are summarized in a recent review [108]. Responding to the urgent medical need of novel drugs for chronic Hepatitis D, Lonafarnib and Myrcludex B received orphan drug status by the European Medicines Agency (EMA) and the U.S. Food and Drug Administration (FDA). Lonafarnib received “Fast Track Status” by the FDA in 2015. Myrcludex B received “prime eligibility status” by the EMA in May 2017.

#### 5.3.1. Lonafarnib

Lonafarnib prevents farnesylation of the *C*-terminal Cys211 residue in the L-HDAg through inhibition of farnesyl transferase, a key enzyme in the prenylation pathway [30,116]. The subsequent blockade of RNP-particle envelopment [117] results in intra-hepatocellular accumulation of HDV replicative intermediates, without shutting down replication *per se*. Thus, the reduction of HDV serum RNA-levels is directly related to HDV assembly (similar to the effect of a NUC for HBV release) and does not primarily lead to a reduction of infected hepatocytes in the liver. However, one effect of Lonafarnib could be accelerated turnover of infected hepatocytes through direct cytotoxic effects of the accumulating replicative intermediates or enhanced immune mediated killing of cells. Such possible modes of action have to be verified in future experiments. Preclinical efficacy of Lonafarnib has been demonstrated in vitro and in mouse models [63,118]. Since Lonafarnib inactivates an essential cellular enzyme [119] and influences important cellular signaling pathways (e.g., the farnesylation of molecules like c-Ras) a particular concern is the long-term tolerability in patients. Initially developed as an anti-cancer drug Lonafarnib has been explored in several clinical phase I and phase II trials [120,121]. A pronounced dose dependent reduction of HDV serum RNA levels has been described in a short-term 4-week study [109]. This demonstrates the efficacy of the drug in vivo, however pronounced adverse events related to drug toxicity were described (weight loss, gastrointestinal side effects.). This led to combination with ritonavir, an inhibitor of CYP3A4, which results in higher local concentrations of the drug in the liver. In this setting, Lonafarnib was tolerated longer leading to reduction of viral loads up to 3.2 log_10_ [110]. In a subsequent still ongoing trial (LOWR HDV-2), patients receive further reduced doses of Lonafarnib (75 mg, 50 mg or 25 mg twice a day) in combination with Ritonavir and with or without pegIFNα for 12–24 weeks. Regarding the virological responses the triple therapy with 25 mg Lonafarnib, 100 mg Ritonavir and pegIFNα showed HDV RNA reduction below the limit of quantification in 5/5 patients at week 24. It will be interesting to see the course of HDV RNA levels during follow up and whether a sustained response after withdrawal of the drug can be observed. It will further be challenging to clinically manage the side effects associated with Lonafarnib, in the event that “functional cure” cannot be achieved and indefinite treatment durations are required.

#### 5.3.2. Nucleic Acid Polymers (REP2139Ca)

Nucleic acid polymers (NAPs) represent a diverse group of oligonucleotides that have been described as agents with a broad spectrum of antimicrobial activity, including inhibition of HIV, HSV or LCMV [115]. The initially proposed mode of action was the interference of NAPs with molecules involved in promoting virus entry (e.g., heparan sulfate proteoglycans). Regarding their antiviral activity against hepadnaviruses, selected NAPs were tested in the duck hepatitis B virus model system. These studies indicated that one compound (REP2006) affected predominantly viral entry while another compound (REP2055) affected DHBV even after establishment of infection leading to accumulation of the drug in duck hepatocytes and a suppression of duck hepatits B virus S-Ag secretion [122,123]. Based on the results in the DHBV model, the first two clinical trials were performed in HBV mono-infected patients [124]. Since the results of these trials indicated a pronounced reduction of serum HBsAg levels, the NAP REP2139-Ca was tested in chronic HDV-infected patients (REP301). 12 HBV/HDV co-infected individuals received monotherapy of 500 mg REP2139-Ca (i.v. once a week) for 15 weeks, followed by combination of 250 mg of the drug with 180 µg pegIFNα for another 15 weeks. PegIFNα was maintained for another 33 weeks and patients were followed up for 24 weeks. Interim results showed a strong reduction of serum HDV RNA accompanied by a pronounced decline in serum levels of HBsAg. A detailed summary of the presented data can be found in [108,115] and on the web site of the company [125]. Recent in vitro data indicate that the inhibitory effect of selected NAPs on HBV entry is abrogated by modification of the 2′-OH of the ribose by a methyl group. Thus, entry inhibition can be excluded as the mode of action of REP2139 (which is a 2′*O*-methyl ribose derivative) in the HDV trial [126]. Remarkably, the same study did not see inhibition of HBsAg secretion by REP2139 in HBV-infected HepaRG-cells, raising the question on how the drug induces the observed reduction of HBsAg-serum levels in patients.

#### 5.3.3. Myrcludex B

Synthetic lipopeptides that mimic the receptor binding-site within the preS1-domain of the HBV L-protein potently block HBV and HDV infection in cell culture and in a humanized mouse model at sub-nanomolar concentrations [46,127,128,129]. Myrcludex B, the lead substance has been characterized regarding its hepatotropism [13,130,131] and has been shown to specifically bind hNTCP in the liver. Remarkably, Myrcludex B targets NTCP of some other species (e.g., mouse, rat and dog) and accumulates at the sinusoidal membrane of differentiated hepatocytes in vivo after intravenous or sub-cutaneous administrations. It associates in vivo with NTCP with half-life times of about 16 h in mice [130,131] and chimpanzees [38]. Myrcludex B inhibits the bile acid transporter function of NTCP [13,132] and induces an increase of conjugated bile acid levels in humans when administered at doses >3–4 mg/person [133]. The IC_50_ of Myrcludex B for bile salt transport inhibition (~47 nM) and HBV and HDV receptor inactivation (80 pM) differ substantially (>500-fold) indicating that HBV/HDV receptor blockade can be achieved without complete saturation of the transporter with the drug. NTCP knockout mice are viable and show no abnormalities beside a slight retard in growth [134]. Recently, several persons with elevated bile salt levels but without apparent clinical syndromes were identified by chance and a functional knock down of NTCP could be assigned [135,136]. Thus, NTCP is presumably eligible for prolonged treatment durations. Myrcludex B has successfully passed phase I safety studies [137] and entered phase IIa clinical trials (Myr-201) in HBeAg-negative HBV and HBV/HBV co-infected patients [138,139]. In the HBV/HDV sub study including 24 HDV/HBV co-infected patients, Myrcludex B was administered in a low, non-NTCP-saturating dose (2 mg daily) either alone, or in combination with pegIFNα for 24 weeks. PegIFNα alone was applied as a control. Myrcludex B was well tolerated and only a slight increase of bile salts was observed. At week 24 HDV RNA declined by more than 1 log in all arms and became negative in two patients each in the Myrcludex B and pegIFNα control arm. Remarkably, negativation was observed in five of seven evaluable patients of the Myrcludex B/pegIFNα combination arm, indicating a synergistic effect of pegIFNα and Myrcludex B. HBV DNA levels were significantly reduced at week 24 in the Myrcludex B/pegIFNα cohort [138,139]. Two ongoing multicenter studies with higher Myrcludex B doses are currently followed: In the Myr-202 trial (*n* = 120) Myrcludex B is administered for 24 weeks at 3 different doses (2 mg, 5 mg and 10 mg) in combination with tenofovir versus tenofovir alone. The second study (Myr-203) combines two doses of Myrcludex B (2 mg and 5 mg) with IFNα for 48 weeks versus Myrcludex B or IFNα alone.

## 6. Conclusions

The re-interest of academic institutions and pharmaceutical companies in HBV research aiming at a better understanding of HBV molecular biology and consequently leading to improved therapies for chronic Hepatitis B also affected HDV research. The availability of in vitro infection systems (as diverse as robust NTCP-expressing hepatoma cell lines for high throughput screening approaches or stem-cell derived hepatocytes to study replication under conditions close to primary hepatocytes) will soon lead to new compounds with novel modes of action on HBV and eventually also on HDV. Available and NTCP-based future animal models will allow verifying and optimizing the activity of these compounds in vivo. Application of these novel systems will improve our understanding on HDV host interactions (e.g., innate immune activation, host factors dependency required for or counteracting against HDV replication, role of the adaptive immune system in elimination of infected cells etc.) in the future

Finally, the already improved state of drug development for chronic HDV-infected patients and the awareness of authorities like the EMA and the FDA for the high medical need to make such drugs instantly available to patients raises a reasonable hope that specific treatment regimens will get approval within the near future. However, it remains a key question whether elimination of HDV can be achieved. In the best case this would be accomplished by elimination or at least control of HBsAg-producing cells, (including those that may express HBsAg from integrates without replicating HBV). Whether this goal can be reached within the coming years is questionable. Thus, the results of the ongoing and still upcoming trials will teach us whether sustained repression of HDV replication can be achieved within a reasonable period of treatment (“functional cure”) or whether long-term therapy with tolerable drug regimens (as currently performed with NUCs in chronic HBV-infected patients) will be required.

## Figures and Tables

**Table 1:** Characteristics of Hepatitis B and Delta Viruses.

	Hepatitis B Virus (HBV)	Hepatitis Delta Virus (HDV)
**Family** **Genus**	*Hepadnaviridae* *Orthohepadnaviruses*	Unassigned *Deltavirus*
**Genome**	relaxed circular partially double-stranded DNA ca. 3.2 kbp	single-stranded (−) RNA 1.7 kb
**Virus-Encoded Proteins**	HBcAg, HBeAg, pol, HBx, L-/M-/S-HBsAg	L-/S-HDAg
**Cellular Receptors**	HSPG, hNTCP	HSPG, hNTCP
**Chronically Infected Individuals Worldwide**	240 million	15–20 million (co-infected with HBV)
**Vaccine Available**	Yes	HBV vaccine
**Curative Therapy Available**	No	No

HBcAg: Hepatitis B core antigen; HBeAg: Hepatitis B e antigen; HBsAg: Hepatitis B surface antigen; pol: polymerase; HBx: Hepatitis B x protein; HDAg: Hepatitis Delta antigen; HSPG: heparan sulfate proteoglycan; hNTCP: human sodium taurocholate cotransporting polypeptide.

**Table 2:** In vitro models for HDV infection.

Model	Entry	Repli-Cation	Progeny Release	Pros	Cons	Ref.
Transfected HuH7	−	+	+ ^1^	Easy access/handling	Does not reflect authentic infection	[42,43,44,45]
PHH	+	+	+ ^2^	Natural host; most physiological	Limited availability; High donor-to-donor variability	[39,40,41]
HepaRG	+	+	+ ^2^	Exhibit some hepatic function; fully support innate immunity	Requires elaborate differentiation protocol; only hepatocyte-like cells are susceptible	[46]
HuH7/HepG2-hNTCP	+	+	+ ^2^	Easy access/handling; efficient infection	Only partially resemble hepatocytes	[13]
Stem-cell derived hepatocytes	+	+	?	Same donor can be used for different experiments; physiologically close to PHH	Requires elaborate differentiation protocol; ethical concerns in some countries	[47,48,49,50,51]

^1^ when co-transfected with a plasmid encoding HBsAg; ^2^ when co-infected with HBV. PHH: primary human hepatocytes.

**Table 3:** In vivo models for HDV infection.

Model	Entry	HBV Co-Infection	Immuno-Competent	Pros	Cons	Ref.
Chimpanzee	+	+	+	Immunocompetent infection model	Ethical considerations	[54,55,56]
Woodchuck	+/-	+ ^1^	+	Immunocompetent infection model	Relies on WHV rather than HBV envelope	[57,59,69]
HDV/HDAg-transgenic mice	-	-	+	Stable mouse lines; tissue-specific expression can be analyzed	No virus infection/spread	[60,61]
Hydrodynamic injection	-	-	+	Fast and easy way to deliver nucleic acids to the liver	No virus infection/spread; harmful to the animal	[62,63]
AAV-HDV transduction	-	-	+	Allows studies of host virus interactions in vivo	No authentic infection system	[70]
Liver-chimeric mice	+	+	-	Authentic HBV/HDV infection; allows for long-term infections	No adaptive immunity; very sophisticated model	[66,67]
hNTCP mice	+	-	+	Immunocompetent transgenic infection system	Low infection rates, transient infection	[64,65]
Macaque/pig hNTCP-transduced	+	+	+	Immunocompetent models allowing authentic infection	Only in vitro data available so far; sophisticated animal models	[15,71]

^1^ Co-infection only with WHV, not with HBV. WHV: Woodchuck Hepatitis Virus.

**Table 4:** Overview of the three novel antiviral drugs in clinical phase II development.

Drug	Target	Mechanism	Clinical Trial Identifier(s)	Company
Lonafarnib	Farnesyl transferase	Assembly inhibition	NCT02430181 NCT02430194	Eiger Bio (Palo Alto, CA, USA)
Nucleic acid polymers	HBsAg?	HDV release inhibition?	NCT02233075 NCT02876419	Replicor (Montreal, QC, Canada)
Myrcludex B	hNTCP	Entry inhibition	NCT02637999	Myr GmbH (Burgwedel, NI, Germany)

## References

[1] Rizzetto M., Canese M.G., Arico S., Crivelli O., Trepo C., Bonino F., Verme G. (1977). Immunofluorescence detection of new antigen-antibody system (delta/anti-delta) associated to hepatitis B virus in liver and in serum of HBsAg carriers. Gut.

[2] Fernandez-Montero J.V., Vispo E., Barreiro P., Sierra-Enguita R., de Mendoza C., Labarga P., Soriano V. (2014). Hepatitis delta is a major determinant of liver decompensation events and death in HIV-infected patients. Clin. Infect. Dis. Off. Publ. Infect. Dis. Soc. Am..

[3] Soriano V., Sherman K.E., Barreiro P. (2017). Hepatitis delta and HIV infection. Aids.

[4] Chen P.J., Kalpana G., Goldberg J., Mason W., Werner B., Gerin J., Taylor J. (1986). Structure and replication of the genome of the hepatitis delta virus. Proc. Natl. Acad. Sci. USA.

[5] Kos A., Dijkema R., Arnberg A.C., van der Meide P.H., Schellekens H. (1986). The hepatitis delta (δ) virus possesses a circular RNA. Nature.

[6] Wang K.S., Choo Q.L., Weiner A.J., Ou J.H., Najarian R.C., Thayer R.M., Mullenbach G.T., Denniston K.J., Gerin J.L., Houghton M. (1986). Structure, sequence and expression of the hepatitis delta (δ) viral genome. Nature.

[7] He L.F., Ford E., Purcell R.H., London W.T., Phillips J., Gerin J.L. (1989). The size of the hepatitis delta agent. J. Med. Virol..

[8] Lamas Longarela O., Schmidt T.T., Schoneweis K., Romeo R., Wedemeyer H., Urban S., Schulze A. (2013). Proteoglycans act as cellular hepatitis delta virus attachment receptors. PLoS ONE.

[9] Leistner C.M., Gruen-Bernhard S., Glebe D. (2008). Role of glycosaminoglycans for binding and infection of hepatitis B virus. Cell. Microbiol..

[10] Schulze A., Gripon P., Urban S. (2007). Hepatitis b virus infection initiates with a large surface protein-dependent binding to heparan sulfate proteoglycans. Hepatology.

[11] Sureau C., Salisse J. (2013). A conformational heparan sulfate binding site essential to infectivity overlaps with the conserved hepatitis B virus A-determinant. Hepatology.

[12] Verrier E.R., Colpitts C.C., Bach C., Heydmann L., Weiss A., Renaud M., Durand S.C., Habersetzer F., Durantel D., Abou-Jaoude G. (2016). A targeted functional RNA interference screen uncovers glypican 5 as an entry factor for hepatitis b and d viruses. Hepatology.

[13] Ni Y., Lempp F.A., Mehrle S., Nkongolo S., Kaufman C., Falth M., Stindt J., Koniger C., Nassal M., Kubitz R. (2014). Hepatitis B and D viruses exploit sodium taurocholate co-transporting polypeptide for species-specific entry into hepatocytes. Gastroenterology.

[14] Yan H., Zhong G., Xu G., He W., Jing Z., Gao Z., Huang Y., Qi Y., Peng B., Wang H. (2012). Sodium taurocholate cotransporting polypeptide is a functional receptor for human hepatitis B and D virus. Elife.

[15] Lempp F.A., Wiedtke E., Qu B., Roques P., Chemin I., Vondran F.W., Le Grand R., Grimm D., Urban S. (2017). Sodium taurocholate cotransporting polypeptide is the limiting host factor of hepatitis B virus infection in macaque and pig hepatocytes. Hepatology.

[16] Li H., Zhuang Q., Wang Y., Zhang T., Zhao J., Zhang Y., Zhang J., Lin Y., Yuan Q., Xia N. (2014). HBV life cycle is restricted in mouse hepatocytes expressing human NTCP. Cell. Mol. Immunol..

[17] Yan H., Peng B., He W., Zhong G., Qi Y., Ren B., Gao Z., Jing Z., Song M., Xu G. (2013). Molecular determinants of hepatitis B and D virus entry restriction in mouse sodium taurocholate cotransporting polypeptide. J. Virol..

[18] Chou H.C., Hsieh T.Y., Sheu G.T., Lai M.M. (1998). Hepatitis delta antigen mediates the nuclear import of hepatitis delta virus RNA. J. Virol..

[19] Tavanez J.P., Cunha C., Silva M.C., David E., Monjardino J., Carmo-Fonseca M. (2002). Hepatitis delta virus ribonucleoproteins shuttle between the nucleus and the cytoplasm. RNA.

[20] Chang J., Nie X., Chang H.E., Han Z., Taylor J. (2008). Transcription of hepatitis delta virus RNA by RNA polymerase II. J. Virol..

[21] Greco-Stewart V.S., Miron P., Abrahem A., Pelchat M. (2007). The human RNA polymerase II interacts with the terminal stem-loop regions of the hepatitis delta virus RNA genome. Virology.

[22] Kuo M.Y., Sharmeen L., Dinter-Gottlieb G., Taylor J. (1988). Characterization of self-cleaving RNA sequences on the genome and antigenome of human hepatitis delta virus. J. Virol..

[23] Wu H.N., Lin Y.J., Lin F.P., Makino S., Chang M.F., Lai M.M. (1989). Human hepatitis delta virus RNA subfragments contain an autocleavage activity. Proc. Natl. Acad. Sci. USA.

[24] Gudima S., Wu S.Y., Chiang C.M., Moraleda G., Taylor J. (2000). Origin of hepatitis delta virus mRNA. J. Virol..

[25] Hsieh S.Y., Chao M., Coates L., Taylor J. (1990). Hepatitis delta virus genome replication: A polyadenylated mRNA for delta antigen. J. Virol..

[26] Lo K., Hwang S.B., Duncan R., Trousdale M., Lai M.M. (1998). Characterization of mRNA for hepatitis delta antigen: Exclusion of the full-length antigenomic RNA as an mRNA. Virology.

[27] Luo G.X., Chao M., Hsieh S.Y., Sureau C., Nishikura K., Taylor J. (1990). A specific base transition occurs on replicating hepatitis delta virus RNA. J. Virol..

[28] Polson A.G., Bass B.L., Casey J.L. (1996). RNA editing of hepatitis delta virus antigenome by dsRNA-adenosine deaminase. Nature.

[29] Wong S.K., Lazinski D.W. (2002). Replicating hepatitis delta virus RNA is edited in the nucleus by the small form of ADAR1. Proc. Natl. Acad. Sci. USA.

[30] Glenn J.S., Watson J.A., Havel C.M., White J.M. (1992). Identification of a prenylation site in delta virus large antigen. Science.

[31] Otto J.C., Casey P.J. (1996). The hepatitis delta virus large antigen is farnesylated both in vitro and in animal cells. J. Biol. Chem..

[32] Hwang S.B., Lai M.M. (1993). Isoprenylation mediates direct protein-protein interactions between hepatitis large delta antigen and hepatitis B virus surface antigen. J. Virol..

[33] Hwang S.B., Lai M.M. (1994). Isoprenylation masks a conformational epitope and enhances trans-dominant inhibitory function of the large hepatitis delta antigen. J. Virol..

[34] Huang H.C., Lee C.P., Liu H.K., Chang M.F., Lai Y.H., Lee Y.C., Huang C. (2016). Cellular nuclear export factors TAP and ALY are required for HDAG-l-mediated assembly of hepatitis delta virus. J. Biol. Chem..

[35] Freitas N., Cunha C., Menne S., Gudima S.O. (2014). Envelope proteins derived from naturally integrated hepatitis B virus DNA support assembly and release of infectious hepatitis delta virus particles. J. Virol..

[36] Sureau C., Guerra B., Lanford R.E. (1993). Role of the large hepatitis B virus envelope protein in infectivity of the hepatitis delta virion. J. Virol..

[37] Sureau C., Guerra B., Lee H. (1994). The middle hepatitis B virus envelope protein is not necessary for infectivity of hepatitis delta virus. J. Virol..

[38] Lempp F.A., Ni Y., Urban S. (2016). Hepatitis delta virus: Insights into a peculiar pathogen and novel treatment options. Nat. Rev. Gastroenterol. Hepatol..

[39] Freitas N., Abe K., Cunha C., Menne S., Gudima S.O. (2014). Support of the infectivity of hepatitis delta virus particles by the envelope proteins of different genotypes of hepatitis B virus. J. Virol..

[40] Gudima S., He Y., Meier A., Chang J., Chen R., Jarnik M., Nicolas E., Bruss V., Taylor J. (2007). Assembly of hepatitis delta virus: Particle characterization, including the ability to infect primary human hepatocytes. J. Virol..

[41] Jaoude G.A., Sureau C. (2005). Role of the antigenic loop of the hepatitis B virus envelope proteins in infectivity of hepatitis delta virus. J. Virol..

[42] Kuo M.Y., Chao M., Taylor J. (1989). Initiation of replication of the human hepatitis delta virus genome from cloned DNA: Role of delta antigen. J. Virol..

[43] Glenn J.S., Taylor J.M., White J.M. (1990). In vitro-synthesized hepatitis delta virus RNA initiates genome replication in cultured cells. J. Virol..

[44] Sureau C., Moriarty A.M., Thornton G.B., Lanford R.E. (1992). Production of infectious hepatitis delta virus in vitro and neutralization with antibodies directed against hepatitis B virus pre-S antigens. J. Virol..

[45] Wu J.C., Chen P.J., Kuo M.Y., Lee S.D., Chen D.S., Ting L.P. (1991). Production of hepatitis delta virus and suppression of helper hepatitis B virus in a human hepatoma cell line. J. Virol..

[46] Gripon P., Rumin S., Urban S., Le Seyec J., Glaise D., Cannie I., Guyomard C., Lucas J., Trepo C., Guguen-Guillouzo C. (2002). Infection of a human hepatoma cell line by hepatitis B virus. Proc. Natl. Acad. Sci. USA.

[47] Kaneko S., Kakinuma S., Asahina Y., Kamiya A., Miyoshi M., Tsunoda T., Nitta S., Asano Y., Nagata H., Otani S. (2016). Human induced pluripotent stem cell-derived hepatic cell lines as a new model for host interaction with hepatitis B virus. Sci. Rep..

[48] Ni Y., Urban S. (2017). Stem cell-derived hepatocytes: A promising novel tool to study hepatitis B virus infection. J. Hepatol..

[49] Sakurai F., Mitani S., Yamamoto T., Takayama K., Tachibana M., Watashi K., Wakita T., Iijima S., Tanaka Y., Mizuguchi H. (2017). Human induced-pluripotent stem cell-derived hepatocyte-like cells as an in vitro model of human hepatitis B virus infection. Sci. Rep..

[50] Shlomai A., Schwartz R.E., Ramanan V., Bhatta A., de Jong Y.P., Bhatia S.N., Rice C.M. (2014). Modeling host interactions with hepatitis B virus using primary and induced pluripotent stem cell-derived hepatocellular systems. Proc. Natl. Acad. Sci. USA.

[51] Xia Y., Carpentier A., Cheng X., Block P.D., Zhao Y., Zhang Z., Protzer U., Jake Liang T. (2016). Human stem cell-derived hepatocytes as a model for hepatitis B virus infection, spreading and virus-host interactions. J. Hepatol..

[52] Alfaiate D., Lucifora J., Abeywickrama-Samarakoon N., Michelet M., Testoni B., Cortay J.C., Sureau C., Zoulim F., Deny P., Durantel D. (2016). Hdv RNA replication is associated with HBV repression and interferon-stimulated genes induction in super-infected hepatocytes. Antivir. Res..

[53] Lempp F.A., Nußbaum L., Rieble L., Ni Y., Urban S. (2016). Screening an FDA-approved drug library on a cell line that supports the full lifecycle of hepatitis deltavirus. J. Hepatol..

[54] Rizzetto M., Canese M.G., Gerin J.L., London W.T., Sly D.L., Purcell R.H. (1980). Transmission of the hepatitis B virus-associated delta antigen to chimpanzees. J. Infect. Dis..

[55] Negro F., Bergmann K.F., Baroudy B.M., Satterfield W.C., Popper H., Purcell R.H., Gerin J.L. (1988). Chronic hepatitis D virus (HDV) infection in hepatitis b virus carrier chimpanzees experimentally superinfected with HDV. J. Infect. Dis..

[56] Sureau C., Taylor J., Chao M., Eichberg J.W., Lanford R.E. (1989). Cloned hepatitis delta virus cDNA is infectious in the chimpanzee. J. Virol..

[57] Ponzetto A., Cote P.J., Popper H., Hoyer B.H., London W.T., Ford E.C., Bonino F., Purcell R.H., Gerin J.L. (1984). Transmission of the hepatitis B virus-associated delta agent to the eastern Woodchuck. Proc. Natl. Acad. Sci. USA.

[58] Fu L., Hu H., Liu Y., Jing Z., Li W. (2017). Woodchuck sodium taurocholate cotransporting polypeptide supports low-level hepatitis B and D virus entry. Virology.

[59] Fiedler M., Kosinska A., Schumann A., Brovko O., Walker A., Lu M., Johrden L., Mayer A., Wildner O., Roggendorf M. (2013). Prime/boost immunization with DNA and adenoviral vectors protects from hepatitis D virus (HDV) infection after simultaneous infection with HDV and Woodchuck hepatitis virus. J. Virol..

[60] Guilhot S., Huang S.N., Xia Y.P., La Monica N., Lai M.M., Chisari F.V. (1994). Expression of the hepatitis delta virus large and small antigens in transgenic mice. J. Virol..

[61] Polo J.M., Jeng K.S., Lim B., Govindarajan S., Hofman F., Sangiorgi F., Lai M.M. (1995). Transgenic mice support replication of hepatitis delta virus RNA in multiple tissues, particularly in skeletal muscle. J. Virol..

[62] Chang J., Sigal L.J., Lerro A., Taylor J. (2001). Replication of the human hepatitis delta virus genome is initiated in mouse hepatocytes following intravenous injection of naked DNA or RNA sequences. J. Virol..

[63] Bordier B.B., Ohkanda J., Liu P., Lee S.Y., Salazar F.H., Marion P.L., Ohashi K., Meuse L., Kay M.A., Casey J.L. (2003). In vivo antiviral efficacy of prenylation inhibitors against hepatitis delta virus. J. Clin. Investig..

[64] He W., Cao Z., Mao F., Ren B., Li Y., Li D., Li H., Peng B., Yan H., Qi Y. (2016). Modification of three amino acids in sodium taurocholate cotransporting polypeptide renders mice susceptible to infection with hepatitis D virus in vivo. J. Virol..

[65] He W., Ren B., Mao F., Jing Z., Li Y., Liu Y., Peng B., Yan H., Qi Y., Sun Y. (2015). Hepatitis D virus infection of mice expressing human sodium taurocholate co-transporting polypeptide. PLoS Pathog..

[66] Dandri M., Lutgehetmann M. (2014). Mouse models of hepatitis B and delta virus infection. J. Immunol. Methods.

[67] Giersch K., Helbig M., Volz T., Allweiss L., Mancke L.V., Lohse A.W., Polywka S., Pollok J.M., Petersen J., Taylor J. (2014). Persistent hepatitis D virus mono-infection in humanized mice is efficiently converted by hepatitis B virus to a productive co-infection. J. Hepatol..

[68] Giersch K., Allweiss L., Volz T., Helbig M., Bierwolf J., Lohse A.W., Pollok J.M., Petersen J., Dandri M., Lutgehetmann M. (2015). Hepatitis delta co-infection in humanized mice leads to pronounced induction of innate immune responses in comparison to HBV mono-infection. J. Hepatol..

[69] Casey J.L., Gerin J.L. (2006). The woodchuck model of HDV infection. Curr. Top. Microbiol. Immunol..

[70] Suarez-Amaran L., Usai C., Di Scala M., Godoy C., Ni Y., Hommel M., Palomo L., Segura V., Olague C., Vales A. (2017). A new HDV mouse model identifies mitochondrial antiviral signaling protein (MAVS) as a key player in IFN-β induction. J. Hepatol..

[71] Protzer U. (2017). Viral hepatitis: The bumpy road to animal models for HBV infection. Nat. Rev. Gastroenterol. Hepatol..

[72] Francois-Souquiere S., Makuwa M., Bisvigou U., Kazanji M. (2016). Epidemiological and molecular features of hepatitis B and hepatitis delta virus transmission in a remote rural community in central Africa. Infect. Genet. Evol..

[73] Ramia S., Bahakim H. (1988). Perinatal transmission of hepatitis B virus-associated hepatitis D virus. Ann. Inst. Pasteur Virol..

[74] Rizzetto M., Ciancio A. (2012). Epidemiology of hepatitis D. Semin. Liver Dis..

[75] Chen X., Oidovsambuu O., Liu P., Grosely R., Elazar M., Winn V.D., Fram B., Boa Z., Dai H., Dashtseren B. (2016). A novel quantitative microarray antibody capture (q-MAC) assay identifies an extremely high HDV prevalence amongst HBV infected mongolians. Hepatology.

[76] Smedile A., Lavarini C., Farci P., Arico S., Marinucci G., Dentico P., Giuliani G., Cargnel A., Del Vecchio Blanco C., Rizzetto M. (1983). Epidemiologic patterns of infection with the hepatitis B virus-associated delta agent in Italy. Am. J. Epidemiol..

[77] Brancaccio G., Giuberti T., Verucchi G., Levantesi M., Sacchini D., Fattovich G., Madonia S., Fasano M., Gavrila C., Nardi A. Epidemiological evolution of chronic hepatitis delta in italy. An analysis of the master-B cohort. Dig. Liver Dis..

[78] Heidrich B., Deterding K., Tillmann H.L., Raupach R., Manns M.P., Wedemeyer H. (2009). Virological and clinical characteristics of delta hepatitis in central Europe. J. Viral Hepat..

[79] Alfaiate D., Deny P., Durantel D. (2015). Hepatitis delta virus: From biological and medical aspects to current and investigational therapeutic options. Antivir.Res..

[80] Gish R.G., Yi D.H., Kane S., Clark M., Mangahas M., Baqai S., Winters M.A., Proudfoot J., Glenn J.S. (2013). Coinfection with hepatitis B and D: Epidemiology, prevalence and disease in patients in northern California. J. Gastroenterol. Hepatol..

[81] Kushner T., Serper M., Kaplan D.E. (2015). Delta hepatitis within the veterans affairs medical system in the United States: Prevalence, risk factors, and outcomes. J. Hepatol..

[82] Liao B., Zhang F., Lin S., He H., Liu Y., Zhang J., Xu Y., Yi J., Chen Y., Liu H. (2014). Epidemiological, clinical and histological characteristics of HBV/HDV co-infection: A retrospective cross-sectional study in Guangdong, China. PLoS ONE.

[83] Shen L., Gu Y., Sun L., Yang Y., Wang F., Li Y., Bi S. (2012). Development of a hepatitis delta virus antibody assay for study of the prevalence of HDV among individuals infected with hepatitis B virus in China. J. Med. Virol..

[84] Hao L.J., Li L., Zhang Y.Y., Song P.H. (1992). Hepatitis D virus infection in liver tissues of patients with hepatitis B in china. Chin. Med. J. (Engl.).

[85] Zhang J.Y., Jin Z.H., Wang C.J. (1995). A seroepidemiological study on hepatitis D virus (HDV) infection in Henan province, China. Zhonghua Liu Xing Bing Xue Za Zhi.

[86] Te H.S., Jensen D.M. (2010). Epidemiology of hepatitis B and C viruses: A global overview. Clin. Liver Dis..

[87] Abe K., Hayakawa E., Sminov A.V., Rossina A.L., Ding X., Huy T.T., Sata T., Uchaikin V.F. (2004). Molecular epidemiology of hepatitis B, C, D and E viruses among children in Moscow, Russia. J. Clin. Virol..

[88] Kozhanova T.V., Il’chenko L., Mikhailov M.I. (2014). Viral hepatitis delta. Is there the delta infection problem in the Russian Federation?. Eksp. Naia Klin. Gastroenterol. Exp. Clin. Gastroenterol..

[89] Semenov A.V. (2012). Prevalence of seronegative hepatitis D among patients with chronic viral hepatitis B. Zhurnal Mikrobiol. Epidemiol. I Immunobiol..

[90] Manesis E.K., Vourli G., Dalekos G., Vasiliadis T., Manolaki N., Hounta A., Koutsounas S., Vafiadis I., Nikolopoulou G., Giannoulis G. (2013). Prevalence and clinical course of hepatitis delta infection in Greece: A 13-year prospective study. J. Hepatol..

[91] El Bouzidi K., Elamin W., Kranzer K., Irish D.N., Ferns B., Kennedy P., Rosenberg W., Dusheiko G., Sabin C.A., Smith B.C. (2015). Hepatitis delta virus testing, epidemiology and management: A multicentre cross-sectional study of patients in London. J. Clin. Virol..

[92] Rizzetto M., Rosina F., Saracco G., Bellando P.C., Actis G.C., Bonino F., Smedile A., Trinchero P., Sansalvadore F., Pintus C. (1986). Treatment of chronic delta hepatitis with α-2 recombinant interferon. J. Hepatol..

[93] Wedemeyer H., Yurdaydin C., Dalekos G.N., Erhardt A., Cakaloglu Y., Degertekin H., Gurel S., Zeuzem S., Zachou K., Bozkaya H. (2011). Peginterferon plus adefovir versus either drug alone for hepatitis delta. N. Engl. J. Med..

[94] Lampertico P., Agarwal K., Berg T., Buti M., Janssen H.L.A., Papatheodoridis G., Zoulim F., Tacke F. (2017). EASL 2017 clinical practice guidelines on the management of hepatitis B virus infection. J. Hepatol..

[95] Soriano V., Vispo E., Sierra-Enguita R., Mendoza C., Fernandez-Montero J.V., Labarga P., Barreiro P. (2014). Efficacy of prolonged tenofovir therapy on hepatitis delta in HIV-infected patients. AIDS.

[96] Hughes S.A., Wedemeyer H., Harrison P.M. (2011). Hepatitis delta virus. Lancet.

[97] Wedemeyer H., Manns M.P. (2010). Epidemiology, pathogenesis and management of hepatitis D: Update and challenges ahead. Nat. Rev. Gastroenterol. Hepatol..

[98] Wranke A., Heidrich B., Hardtke S., Wedemeyer H. (2015). Current management of HBV/HDV coinfection and future perspectives. Curr. Hepatol. Rep..

[99] Zoulim F., Carosi G., Greenbloom S., Mazur W., Nguyen T., Jeffers L., Brunetto M., Yu S., Llamoso C. (2015). Quantification of HBsAg in nucleos(t)ide-naive patients treated for chronic hepatitis B with entecavir with or without tenofovir in the be-low study. J. Hepatol..

[100] Heidrich B., Yurdaydin C., Kabacam G., Ratsch B.A., Zachou K., Bremer B., Dalekos G.N., Erhardt A., Tabak F., Yalcin K. (2014). Late HDV RNA relapse after peginterferon α-based therapy of chronic hepatitis delta. Hepatology.

[101] Wedemeyer H., Yurdaydin C., Ernst S., Caruntu F.A., Curescu M.G., Yalcin K., Akarca U.S., Gürel S., Zeuzem S., Erhardt A. (2014). O4 prolonged therapy of hepatitis delta for 96 weeks with pegylated-interferon-α-2A plus tenofovir or placebo does not prevent HDV RNA relapse after treatment: The HIDIT-2 study. J. Hepatol..

[102] Lai M.M. (2005). RNA replication without RNA-dependent RNA polymerase: Surprises from hepatitis delta virus. J. Virol..

[103] Yen L., Magnier M., Weissleder R., Stockwell B.R., Mulligan R.C. (2006). Identification of inhibitors of ribozyme self-cleavage in mammalian cells via high-throughput screening of chemical libraries. RNA.

[104] Wooddell C.I., Rozema D.B., Hossbach M., John M., Hamilton H.L., Chu Q., Hegge J.O., Klein J.J., Wakefield D.H., Oropeza C.E. (2013). Hepatocyte-targeted RNAi therapeutics for the treatment of chronic hepatitis B virus infection. Mol. Ther..

[105] Yuen M.F., Chan H.L., Liu S., Given B., Schluep T., Hamilton J., Lai C.L., Locarnini S., Lau J.Y., Ferrari C. (2015). Arc-520 produces deep and durable knockdown of viral antigens and DNA in a phase II study in patients with chronic hepatitis B. Hepatology.

[106] Sureau C., Negro F. (2016). The hepatitis delta virus: Replication and pathogenesis. J. Hepatol.

[107] Zhang Z., Ni Y., Filzmayer C., Urban S. MDA5 mediated activation of innate immune responses by Hepatitis D virus infection. Proceedings of the International Meeting on the Molecular Biology of Hepatitis B Viruses.

[108] Elazar M., Glenn J.S. (2017). Emerging concepts for the treatment of hepatitis delta. Curr. Opin. Virol..

[109] Koh C., Canini L., Dahari H., Zhao X., Uprichard S.L., Haynes-Williams V., Winters M.A., Subramanya G., Cooper S.L., Pinto P. (2015). Oral prenylation inhibition with lonafarnib in chronic hepatitis D infection: A proof-of-concept randomised, double-blind, placebo-controlled phase 2a trial. Lancet. Infect. Dis..

[110] Yurdaydin C., Idilman R., Choong I., Kalkan C., Keskin O., Karakaya M.F., Tuzun A.E., Karatayli E., Bozdayi M., Cory D. (2015). O118: Optimizing the prenylation inhibitor lonafarnib using ritonavir boosting in patients with chronic delta hepatitis. J. Hepatol..

[111] Koh C., Yurdaydin C., Cooper S.L., Cory D., Dahari H., Haynes-Williams V., Winters M., Bys M., Choong I., Idilman R. (2014). Prenylation inhibition with lonafarnib decreases hepatitis D levels in humans. Hepatology.

[112] Bazinet M., Pantea V., Cebotarescu V., Cojuhari L., Jimbei P., Vaillant A. (2015). Hdv2 o-09: Rep 2139 monotherapy and combination therapy with pegylated interferon: Safety and potent reduction of HBsAg and HDV RNA in caucasian patients with chronic HBV/HDV co-infection. J. Viral Hepat..

[113] Poutay D., Sabra M., Abou-Jaoude G., Chemin I., Trepo C., Vaillant A., Sureau C. (2015). P177: Nucleic acid polymers are efficient in blocking hepatitis delta virus entry in vitro. J. Viral Hepat..

[114] Bazinet M., Pantea V., Cebotarescu V., Cojuhari L., Jimbei P., Albrecht J., Schmid P., Karimzadeh H., Roggendorf M., Vaillant A. (2015). Update on the safety and efficacy of rep 2139 mono-therapy and subsequent combination therapy with pegylated interferon α-2a in chronic HBV/HDV co-infection in caucasian patients. Hepatology.

[115] Vaillant A. (2016). Nucleic acid polymers: Broad spectrum antiviral activity, antiviral mechanisms and optimization for the treatment of hepatitis B and hepatitis D infection. Antivir. Res..

[116] Yust-Katz S., Liu D., Yuan Y., Liu V., Kang S., Groves M., Puduvalli V., Levin V., Conrad C., Colman H. (2013). Phase 1/1b study of lonafarnib and temozolomide in patients with recurrent or temozolomide refractory glioblastoma. Cancer.

[117] Rizzetto M., Ciancio A. (2015). The prenylation inhibitor, lonafarnib: A new therapeutic strategy against hepatitis delta. Lancet Infect. Dis..

[118] Bordier B.B., Marion P.L., Ohashi K., Kay M.A., Greenberg H.B., Casey J.L., Glenn J.S. (2002). A prenylation inhibitor prevents production of infectious hepatitis delta virus particles. J. Virol..

[119] Mijimolle N., Velasco J., Dubus P., Guerra C., Weinbaum C.A., Casey P.J., Campuzano V., Barbacid M. (2005). Protein farnesyltransferase in embryogenesis, adult homeostasis, and tumor development. Cancer Cell.

[120] Berndt N., Hamilton A.D., Sebti S.M. (2011). Targeting protein prenylation for cancer therapy. Nat. Rev. Cancer.

[121] Palsuledesai C.C., Distefano M.D. (2015). Protein prenylation: Enzymes, therapeutics, and biotechnology applications. ACS Chem. Biol..

[122] Noordeen F., Scougall C.A., Grosse A., Qiao Q., Ajilian B.B., Reaiche-Miller G., Finnie J., Werner M., Broering R., Schlaak J.F. (2015). Therapeutic antiviral effect of the nucleic acid polymer rep 2055 against persistent duck hepatitis B virus infection. PLoS ONE.

[123] Noordeen F., Vaillant A., Jilbert A.R. (2013). Nucleic acid polymers prevent the establishment of duck hepatitis B virus infection in vivo. Antimicrob. Agents Chemother..

[124] Al-Mahtab M., Bazinet M., Vaillant A. (2016). Safety and efficacy of nucleic acid polymers in monotherapy and combined with immunotherapy in treatment-naive Bangladeshi patients with HBeAg+ chronic hepatitis B infection. PLoS ONE.

[125] Replicor. http://replicor.com/science/conference-presentations/.

[126] Guillot C., Martel N., Berby F., Bordes I., Hantz O., Blanchet M., Sureau C., Vaillant A., Chemin I. (2017). Inhibition of hepatitis B viral entry by nucleic acid polymers in HepaRG cells and primary human hepatocytes. PLoS ONE.

[127] Gripon P., Cannie I., Urban S. (2005). Efficient inhibition of hepatitis B virus infection by acylated peptides derived from the large viral surface protein. J. Virol..

[128] Petersen J., Dandri M., Mier W., Lutgehetmann M., Volz T., von Weizsacker F., Haberkorn U., Fischer L., Pollok J.M., Erbes B. (2008). Prevention of hepatitis B virus infection in vivo by entry inhibitors derived from the large envelope protein. Nat. Biotechnol..

[129] Schulze A., Schieck A., Ni Y., Mier W., Urban S. (2010). Fine mapping of pre-s sequence requirements for hepatitis B virus large envelope protein-mediated receptor interaction. J. Virol..

[130] Meier A., Mehrle S., Weiss T.S., Mier W., Urban S. (2013). Myristoylated PRES1-domain of the hepatitis B virus l-protein mediates specific binding to differentiated hepatocytes. Hepatology.

[131] Schieck A., Schulze A., Gahler C., Muller T., Haberkorn U., Alexandrov A., Urban S., Mier W. (2013). Hepatitis B virus hepatotropism is mediated by specific receptor recognition in the liver and not restricted to susceptible hosts. Hepatology.

[132] Nkongolo S., Ni Y., Lempp F.A., Kaufman C., Lindner T., Esser-Nobis K., Lohmann V., Mier W., Mehrle S., Urban S. (2014). Cyclosporin a inhibits hepatitis B and hepatitis D virus entry by cyclophilin-independent interference with the NTCP receptor. J. Hepatol..

[133] Haag M., Hofmann U., Murdter T.E., Heinkele G., Leuthold P., Blank A., Haefeli W.E., Alexandrov A., Urban S., Schwab M. (2015). Quantitative bile acid profiling by liquid chromatography quadrupole time-of-flight mass spectrometry: Monitoring hepatitis B therapy by a novel Na(+)-taurocholate cotransporting polypeptide inhibitor. Anal. Bioanal. Chem..

[134] Slijepcevic D., Kaufman C., Wichers C.G., Gilglioni E.H., Lempp F.A., Duijst S., de Waart D.R., Elferink R.P., Mier W., Stieger B. (2015). Impaired uptake of conjugated bile acids and hepatitis B virus PRES1-binding in Na(+) -taurocholate cotransporting polypeptide knockout mice. Hepatology.

[135] Vaz F.M., Paulusma C.C., Huidekoper H., de Ru M., Lim C., Koster J., Ho-Mok K., Bootsma A.H., Groen A.K., Schaap F.G. (2015). Sodium taurocholate cotransporting polypeptide (SLC10A1) deficiency: Conjugated hypercholanemia without a clear clinical phenotype. Hepatology.

[136] Deng M., Mao M., Guo L., Chen F.P., Wen W.R., Song Y.Z. (2016). Clinical and molecular study of a pediatric patient with sodium taurocholate cotransporting polypeptide deficiency. Exp. Ther. Med..

[137] Blank A., Markert C., Hohmann N., Carls A., Mikus G., Lehr T., Alexandrov A., Haag M., Schwab M., Urban S. (2016). First-in-human application of the first-in-class hepatitis B and hepatitis D virus entry inhibitor myrcludex B. J. Hepatol..

[138] Bogomolov P., Voronkova N., Allweiss L., Dandri M., Schwab M., Lempp F.A., Haag M., Wedemeyer H., Alexandrov A., Urban S. (2014). A proof-of-concept phase 2a clinical trial with HBV/HDV entry inhibitor myrcludex b. Hepatology.

[139] Bogomolov P., Alexandrov A., Voronkova N., Macievich M., Kokina K., Petrachenkova M., Lehr T., Lempp F.A., Wedemeyer H., Haag M. (2016). Treatment of chronic hepatitis D with the entry inhibitor myrcludex b—First results of a phase Ib/IIa study. J. Hepatol..

